# The Predicative Power of Learner and Teacher Variables on Flow in a Chinese Blended English as a Foreign Language Learning Context

**DOI:** 10.3389/fpsyg.2022.849570

**Published:** 2022-03-22

**Authors:** Xin Wang

**Affiliations:** School of Humanities and Foreign Languages, Xi’an University of Science and Technology, Xi’an, China

**Keywords:** learner-internal variables, teacher-related variables, foreign language flow, blended EFL learning, positive and negative emotions, positive psychology, predicative effects

## Abstract

The “dynamic turn” in the field of second language acquisition catalyzed scholarly devotion to the complex dynamic relationships between learner and teacher variables and various academic emotions. As such, the present study examined the varying effects of the aforementioned variables on the constructs of positive and negative flow, and determined their strongest predictors, respectively. This study used a mixed-method approach to collect data from 607 Chinese English-as-a-Foreign-Language learners. In stage one of the research, the researcher first assessed the participants’ levels of positive flow and negative flow in a blended learning context, then performed Pearson correlation analysis to confirm a significant, but weak positive relationship between positive flow and negative flow. Then, significant difference tests were run to determine the varied effects of those variables on flow. Finally, two multiple regression analyses were conducted to identify five predictors of positive flow, with the biggest contribution coming from learners’ attitudes toward the foreign language, and three predictors of negative flow, with the learners’ major accounting for the majority of variance. In the second stage of the research, a qualitative corpus was constructed, based on accounts of classroom experiences from 71 participants of the total sample, and further illustrated the quantitative findings. Pedagogical implications for educational psychologists and teachers of second and/or foreign languages are addressed.

## Introduction

In the wake of the “dynamic turn” in the field of second language acquisition (SLA) (de Bot et al., 2007; Larsen-Freeman and Cameron, 2008), researchers have increasingly viewed language learning as a complex dynamic system, acknowledging the affective component to language learning (Dörnyei and Ryan, 2015). In the meantime, the last few decades have witnessed an upsurge in research seeking to examine emotions in blended learning (Shin, 2006; Lajoie et al., 2020; Lorderer et al., 2020) which has been shown to be the most effective and efficient combination of traditional face-to-face instruction and online learning (Neumeier, 2005). As such, the academic focus was set firmly and irrevocably on exploring factors likely to promote optimal learning in an affectively favorable technology-rich environment (Astleitner and Leutner, 2014; Lorderer et al., 2018; Wang and Derakhshan, 2021), where control-value appraisals and characteristics of blended learning were confirmed as antecedents for various emotions (Daniels and Stupnisky, 2012; Lorderer et al., 2018).

A powerful conceptualization of a positive emotional experience (Dewaele and MacIntyre, 2019), flow is characterized by individuals’ heightened engagement (Aubrey, 2017) in various tasks and dynamic interactions with the environment (Nakamura and Csíkszentmihályi, 2002). When learners undergo this type of optimal experience, positive results are naturally expected (Schüler, 2012). There is, however, a misunderstanding of the term “optimal,” which actually refers to “the inner state of perfect and mental functioning, but not to the desirability of its outcome” (Schüler, 2012, p. 133). As well, flow theory does not explicitly deny the negative effects of that experience (Csíkszentmihályi, 1990). Indeed, the positive side of flow (PosFlow) is usually accompanied by its negative side (anti-flow or NegFlow) (Csíkszentmihályi and Rathunde, 1993).

Therefore, to revitalize this area, it is important to place a renewed emphasis on the dynamics of flow, exploring how positive flow and negative flow interact in blended learning. This study adopts an explanatory sequential design (cf. Cresswell and Plano Clark, 2018) and empirically examines the predictive effects of learner-internal and teacher-related variables on flow and their correlates with positive and negative flow in a large sample of English as a Foreign Language (EFL) students in a blended learning environment. Further, a corpus of learner feedback supported the aforementioned correlations.

## Literature Review

### Flow Theory

As an umbrella concept of emotions (Csíkszentmihályi, 1990; Dewaele and MacIntyre, 2019), flow refers to a dynamic interaction between an individual and the environment that occurs in the course of completing a task, where individuals are fully engaged, picking up interests, and in turn, enjoying the activity and recognizing it as intrinsically rewarding (Csíkszentmihályi, 2014). Considering the potential role of flow in facilitating intrinsic motivation and expanding the scope of one’s goals and interests (Csíkszentmihályi and Nakamura, 1999), educational psychologists have examined flow from a variety of perspectives, ranging from phenomenological behaviors (Nakamura and Csíkszentmihályi, 2002), complex system of graded challenges and stretched skills (Moneta and Csíkszentmihályi, 1996), to autotelic personality (Csíkszentmihályi, 1975).

Ever since, a series of studies on autotelic personality and activities have shed light on how individuals focused their attention on tasks primarily for their own sake rather than for external rewards (Csíkszentmihályi, 1997). The academic literature gradually moves away from a paradigm of homeostatic equilibrium toward a view of flow experience as a complex dynamic process that interacts with individual differences (Dörnyei and Ryan, 2015), intrinsic motivation (Nakamura and Csíkszentmihályi, 2002), and peak performance (Csíkszentmihályi, 1975, 1990; Csíkszentmihályi and Rathunde, 1993). Moreover, those who experience flow are more likely to be focused and enjoy themselves in their work (Csíkszentmihályi and LeFevre, 1989), art and science (Csíkszentmihályi, 1996), and academic learning (Perry, 1999; Schüler, 2007).

Now that the provoking mechanism of flow is complex and dynamic, researchers have vigorously investigated the antecedents for flow experiences, flow characteristics, and its consequences (Nakamura and Csíkszentmihályi, 2002), contributing to an extensive repertoire of flow-related variables. Specifically, the preconditions of flow were widely accepted to include the perceived challenge-skills balance, clear proximal goals, and immediate feedback (Csíkszentmihályi, 1975; Shin, 2006; Li R. et al., 2021; Liu and Song, 2021), while flow experiences have expanded beyond the original scope of intense focus, emergent action and awareness, loss of self-consciousness, a sense of control, distortion of temporal experience, and perception of intrinsically motivated activities (Csíkszentmihályi, 1975), to include telepresence, engagement (Shin, 2006), and peer interaction (Liu and Song, 2021). Further, flow consequences were mainly manifestated by academic achievement (Carli et al., 1988; Csíkszentmihályi, 1996; Egbert, 2003; Dewaele and MacIntyre, 2019), course satisfaction (Buil et al., 2019; Li R. et al., 2021; Liu and Song, 2021), and change of attitude and behavior (Liu and Song, 2021).

Later, the complex dynamic view catalyzed an expanded model of flow with eight experiential quadrants inside of a concentric circle (Carli et al., 1988), a model that has been confirmed robustly by subsequent studies (Moneta and Csíkszentmihályi, 1996; Csíkszentmihályi, 1997; Dewaele and MacIntyre, 2019). The modified model yielded eight different challenge/skill formulas, which incorporated aversive emotions into the construct of flow and distinguished boredom, apathy, anxiety, and worry from control, relaxation, arousal, and (positive) flow. It is possible, on the one hand, for individuals to fully engage in tasks and enjoy the activity to the extent that their skills and abilities match the task complexity, leading them to the ideal quadrant of positive flow in contrast to the aversive emotion of anxiety, where the task challenge is greater than skills to the extent that people feel unable to cope and become tense. On the other hand, negative flow experiences mainly corresponded with the quadrants of boredom and apathy, with apathy striking the most unfavorable formula where low challenge and skills, though coordinated, yielded a stagnated or diffusive mind (Nakamura and Csíkszentmihályi, 2002).

### Flow and Foreign Language Learning

Language learning is a complex, long-lasting process that involves continuous dynamic interactions between learners, teachers, and the environment (Dörnyei and Ryan, 2015; Wang and Derakhshan, 2021), with learning a foreign or second language (FL/L2) being particularly difficult when task challenge and learners’ proficiency do not always align. On balance, a variety of formulas of challenge and skills generate a wide range of emotions in the FL contexts. As previous research has extensively focused on the disruptive influences of foreign language anxiety, especially foreign language classroom anxiety (FLCA) (Horwitz, 2010; Dewaele and MacIntyre, 2014), positive emotions have instead long been shunned, if not completely ignored, until positive psychologists advocated a holistic view of emotions (Dewaele and MacIntyre, 2014; MacIntyre and Mercer, 2014; MacIntyre et al., 2016) and gradually shifted their attention to positive emotions (MacIntyre and Vinze, 2017; Wang Y. L. et al., 2021). As well, after Csíkszentmihályi (1990) extended flow theory to the field of SLA, flow has substantially contributed to positive psychology (Czimmermann and Piniel, 2016; MacIntyre et al., 2016) and provided a colorful palette of insights into the dynamic interactions between positive and negative emotions beyond the scope of enjoyment and anxiety to include boredom, relaxation, apathy, shame, guilt, and burnout (Teimouri, 2018; Li C. et al., 2021).

Regardless of the holistic view of emotions in SLA, flow has long been recognized as a “highly desirable state associated with a broad variety of positive outcomes in terms of positive motivation, well-being, and performance” (Schüler, 2012, p. 123) and associated exclusively with positive emotions in accordance with Seligman’s (2011) PERMA framework of happiness and well-being. Nonetheless, a state of positive flow is typically characterized by a balance between positive and negative emotions in the mental functioning system of consciousness (Dewaele and MacIntyre, 2019) wherein FL learners’ intense focus contributes to emergent awareness system, which engages learners in selecting, processing and storing information (Nakamura and Csíkszentmihályi, 2002). Since FL learners are easily exposed to failure in a threatening environment (Dörnyei and Ryan, 2015), their ability to focus may highly correlate with either their positive or negative attitudes toward and achievements in the FL classroom, which may be also related to their academic engagement.

However, there is a lack of literature exploring the positive and negative aspects of flow (Czimmermann and Piniel, 2016; Dewaele and MacIntyre, 2019; Wang and Huang, 2022) until Oxford (2016) proposed the EMPATHICS model of well-being and posited that positive emotions coexisted with negative emotions in a person. Since then, a significant body of evidence has begun to confirm the robustness of the juxtaposition of positive flow (PosFlow) and negative flow (anti-flow or NegFlow) (Csíkszentmihályi and Rathunde, 1993), and substantiate a combination of PosFlow and NegFlow in SLA (Dewaele and MacIntyre, 2019). The seminal study was conducted by Czimmermann and Piniel (2016), who explicitly illustrated the concurrence of positive and negative emotions in flow. However, they did not examine individual difference factors likely to provoke positive or negative flow experiences and the dynamic interactions between individual differences and their emotions. After this, Dewaele and MacIntyre (2019) investigated the dynamic relationship between positive and negative flow and revealed the effects of learner-internal (sociodemographic and linguistic) variables on both positive and negative flow. Despite the fact that their study broaden the scope of inquiry, Dewaele and MacIntyre (2019) did overlook the robustness of the global flow measure, as evidenced by a borderline Cronbach reliability coefficient of 0.60 for the negative flow measurement scale, and not look into the learner-external factors (e.g., teacher-centered variables and the learning environment) that may cause oscillations in individual learners’ emotional experiences. In this case, research into the correlations between predicative factors and flow experiences was confined to imprecise assessment of participants’ levels of flow in both physical and online learning settings. To address this gap, Wang and Huang (2022) developed the *Foreign Language Flow Scale* (FLFS) that captures characteristics of a blended EFL learning environment and allows further investigation into the relationships between positive and negative flow and other learner and teacher variables in that environment.

### Flow in a Blended English as a Foreign Language Learning

Blended learning, also known as hybrid learning, refers to the optimal mixing of online and face-to-face learning (Bowyer, 2017) that is likely to improve learners’ learning outcomes and their sense of achievement. Despite this, it should not be ignored that blended learning, though being highly engaging and intriguing (Rafiee and Abbasian-Naghneh, 2021), is subject to the level of learner autonomy, motivation (Wang and Derakhshan, 2021), and learners’ technological self-efficacy (Lorderer et al., 2020; Pan, 2020; Rafiee and Abbasian-Naghneh, 2021), their technology acceptance and readiness (Barrett et al., 2020; Rafiee and Abbasian-Naghneh, 2021), as well as teachers’ willingness to and proficiency in carrying out technology-based FL activities. As a rule, learners’ prior experience with technology-based environments may influence their perceptions of control over a task (Rafiee and Abbasian-Naghneh, 2021), which in turn can engender diverse emotions (Lorderer et al., 2020). As a result, the blended learning may not necessarily lead to positive academic or emotional outcomes. Intricate interactions were confirmed between positive and negative emotions of FL learners in a blended environment (Dewaele and MacIntyre, 2019; Rafiee and Abbasian-Naghneh, 2021; Wang and Huang, 2022) where FL learners may lose control over a FL task and sometimes are not fully engaged in the process (Wang and Huang, 2022).

Positive flow, in general, necessitates a harmonious balance between challenge and skills, clear learning goals, and immediate feedback, all of which are widely acknowledged as its prerequisites. These preconditions are compatible with a framework for blended learning that provides learners with easy access to resources, flexible adaptation of task complexity, and instant feedback (Wang X. et al., 2021). In this way, they facilitate engagement and capture the attention of audiences (Albiladi and Alshareef, 2019). Further, interest in FL learning peaks when learners are intensively engaged in a task, which eventually leads to a sense of confidence in and satisfaction with the blended FL classes (Albiladi and Alshareef, 2019; Rafiee and Abbasian-Naghneh, 2021; Wang and Huang, 2022). In this manner, their positive L2 self is established and integrated into their technological self-efficacy (Yilmaz, 2017; Rafiee and Abbasian-Naghneh, 2021). Consequently, blended EFL learning environment further revitalizes their motivation to learn (Wang and Derakhshan, 2021) and reinforces subsequent FL learning behaviors (Dörnyei and Ryan, 2015), which is aligned with the theoretical framework of the L2 Motivational Self System (Dörnyei, 2009) in which learners are intrinsically motivated by active participation rather than external rewards. Also, the gap between the current self and the future self has narrowed at a proximal challenge-skills balance (Dörnyei, 2009), especially in a blended learning environment where tasks are much more flexible to deliver and adapt, and FL learners are more likely to be satisfied with the FL course and experience the positive flow (Davis et al., 1992; Wang and Huang, 2022). However, as FL learners’ attention tends to drift to things other than learning and perceived their FL learning to be somewhat dull or anxious sometimes, flow experiences are confirmed to be filled with both benefits and challenges in a blended learning environment (Wang and Huang, 2022). Therefore, positive and negative emotions are juxtaposed in the construct of flow both in traditional and blended foreign and/or second language learning milieu, which allows dynamic interactions between individual learner-and-teacher variables, learning environments and learners’ varying dispositions to experience positive or negative flow.

## Research Purpose and Questions

The literature review suggests that, while significant research has been conducted on positive side of flow, it would be worthwhile for further investigation to combine the positive and negative aspects in a blended learning environment. In this study, the researcher proposes that flow-enhancing and flow-inhibiting experiences are indispensable components of flow (Schüler, 2012; Dewaele and MacIntyre, 2019), and that learners’ technology acceptance and readiness influence the process of and subsequent attitudes toward L2 learning (Pan, 2020; Rafiee and Abbasian-Naghneh, 2021). Further, what remains unexplored is to what extent the same learner-internal and teacher-related variables affect both positive and negative flow in a blended learning environment. These are what the present study aims to address.

Research questions: (1) How much flow^1^ do Chinese EFL learners report on the FLFS in a Chinese blended EFL learning context at the tertiary level? (2) What is the relationship between positive flow and negative flow? (3) To what extent are positive flow and negative flow associated with learner-internal variables (i.e., age, gender, major, years spent in learning English, scores in *Gaokao*^2^ and English final examination, students’ self-perceived English proficiency, their estimation of the overall FL mastery, attitude toward the FL, and familiarity with technology use in the FL class) and teacher-related variables (e.g., time spent in instructing writing, reading, listening, speaking, and translating, students’ attitude toward the FL teacher, teacher’s frequency of FL use, and teacher predictability)? (4) How do these significant predictors identified co-predict positive and negative flow within one specific educational context?

## Materials and Methods

### Procedure and Participants

The researcher recruited 607 participants through a convenience sampling procedure. To ensure an unbiased response to the questionnaire, participants were informed of the research goals and procedures. After the researcher obtained written informed consent from the participants, the final version was sent to students via *Wenjuanxing*^3^. All participants came from the same university in Northwest China, a multidisciplinary organization specializing in coal-mining security. The participants in this study were all non-English majors specializing in natural science or social science subjects during their first academic semester of Year 2. The participants’ demographic (e.g., age, gender, major, years spent in learning English, English score in Gaokao English and the most recent final exam) and linguistic information (e.g., students’ self perceived English proficiency) is summarized in Table 1.

**TABLE 1:** Detailed participant information.

Variables	Research stages	Group		Male	Female	NS	SS	Mean (*SD*)				
								Age	YSLE	GKE	SPEP	FS
**FLF**	**Stage 1**	Whole	607	383	224	498	109	19.41 (0.922)	10.46 (2.46)	98.18 (22.13)	2.45 (0.839)	57.12 (26.89)
	**Stage 2**	Whole	71	47	24	71	0	18.94 (0.53)	10.77 (2.04)	103.48 (16.89)	5.04 (1.86)	66.07 (13.82)

*Whole, whole sample; YSLE, years spent in learning English; GKE, English score in Gaokao; FS, score in English final exam in college.*

Regarding the language-related section, the participants first reported their English scores in Chinese *Gaokao*, then rated their perceived language proficiency on a 1-to-10 scale (*M* = 5.01, *SD* = 3.032). In addition, participants’ English results on the most recent final examination were obtained from an official database. Following that, participants were asked to rate their general mastery of FL (ranging from “beginner” to “advanced”) (*M* = 2.36, *SD* = 0.768) and the extent to which they felt that their FL competence exceeded that of their FL classmates (ranging from “far below average” to “far above average”), resulting in a mean score of 2.54 (*SD* = 0.862). Afterward, participants were asked to indicate their level of positive attitude toward the FL they were studying (*M* = 3.24, *SD* = 0.87), and toward the FL teacher (*Mean* = 4.01, *SD* = 0.78) on a 5-point Likert scale. The next question asked participants to indicate how familiar they were with the use of technology in the FL class (ranging from “very unfamiliar” to “very familiar”) (*Mean* = 3.46, *SD* = 0.83). Then, participants were asked how frequently their FL teacher uses the FL in class (*Mean* = 3.79, *SD* = 0.98) and how much time their FL teacher spends instructing them to write, read, listen, speak and translate (the total was 100%). This section concluded with participants asked to indicate on a 5-point Likert scale how predictable the teacher was in a blended learning environment (*Mean* = 3.43, *SD* = 0.74). The Appendix provides further demographic and language-related information.

### The Instrument

In the first stage of the research, quantitative data were collected through a composite questionnaire which started with a sociodemographic and linguistic section inquiring about participants’ backgroup (see Table 1 and Appendix) and the *Foreign Language Flow Scale* asking about positive and negative flow experiences in a Chinese blended learning context.

**FIGURE 1:**
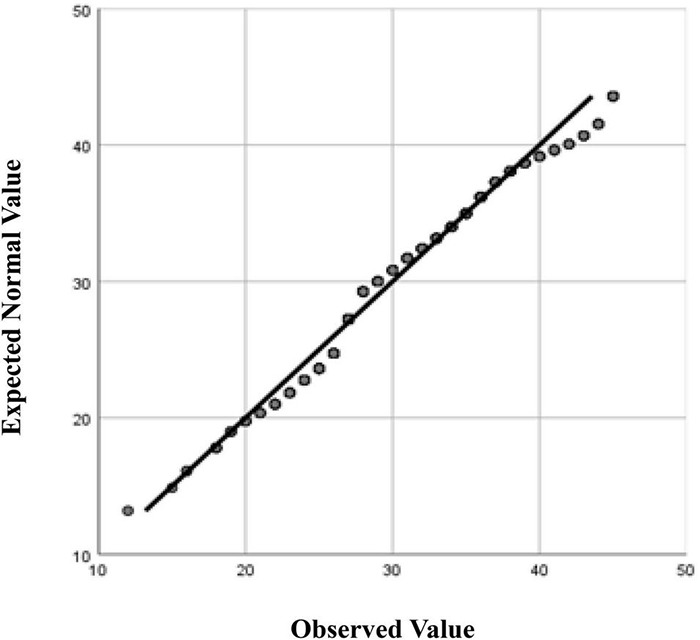
Normal Q-Q Plot of PosFlow.

**FIGURE 2:**
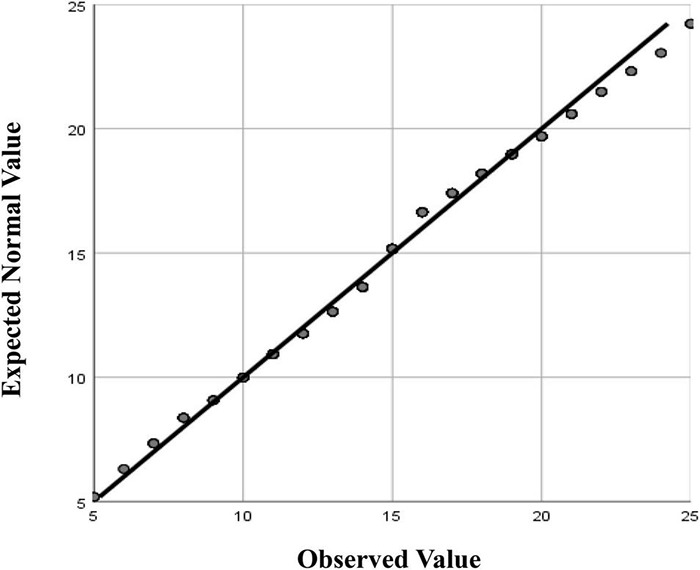
Normal Q-Q Plot of NegFlow.

The 14-item *Foreign Language Flow Scale* (FLFS) was derived from the *Classroom Flow Questionnaire* (CFQ) (Czimmermann and Piniel, 2016) and adapted to better fit the Chinese EFL context in a blended learning environment (Wang and Huang, 2022). The FLFS consists of items that reflect positive (one dimension) and negative (two dimensions) flow experiences: *FLF-Enjoyment*, *FLF-Boredom*, and *FLF-Anxiety*. For FLF 1 to FLF 14, the anchors were “not at all” = 1, “seldom” = 2, “occasionally” = 3, “sometimes” = 4, and “usually” = 5. In order to satisfy its indicator nature as both positive and negative flow, all items were alternately coded either in positive or negative terms. Furthermore, Wang and Huang’s (2022) study confirmed its construct validity [^Δ^χ^2^ (74) = 2.42, CFI = 0.966, TLI = 0.958, RMSEA = 0.044, SRMR = 0.039, AVE = 0.513] and internal consistency (Cronbach α = 0.785, composite reliability = 0.936). In addition, a scale analysis of the FLFS in the current study revealed desirable validity [^Δ^χ^2^ (74) = 2.46, CFI = 0.957, TLI = 0.947, RMSEA = 0.049, SRMR = 0.038, AVE = 0.515] and high internal consistency (Cronbach α = 0.825, composite reliability = 0.936). When examining the distribution of PosFlow and NegFlow scores and calculating Q-Q plots (Figures 1, 2), it can be seen that FL learners’ flow experiences follow a normal distribution reasonably well except for the extreme tail (see Figure 1). Thus, the researcher used the more powerful parametric statistic to address the research questions.

In the second stage of the research, qualitative data were obtained by asking a final open question, “Describe (a) specific event(s) or episode(s) in your FL class in which you really enjoyed and were focused with strong interest, or one in which you were absent-minded, (if not) anxious, and bored, and provide as much information as possible.” Seventy-one out of a total sample answered the question, yielding 65 valid responses and 5998 words. The average length of feedback entries was 92.28 words. The dataset depicted a landscape of FL learners’ flow experiences as described by participants. Table 1 provides the demographic details of participants in the second stage of the research.

### Data Analysis

The researcher adopted an explanatory sequential design. In this design, the researcher conducted descriptive analyses, ANOVAs, and multiple regression analyses using SPSS 26.0 to collect quantitative data in order to (a) assess Chinese EFL learners’ level of flow in a blended learning environment, (b) identify the relationship between positive flow and negative flow, (c) investigate the links between learner and teacher variables, and positive flow and negative flow, and (d) examine the co-predicting effects of the aforementioned variables on positive flow and negative flow. In addition, the researcher conducted a qualitative analysis to further explain the initial quantitative results (Cresswell and Plano Clark, 2018). Furthermore, the qualitative data aims to provide examples of positive and negative flow described by participants themselves. Thus, results from quantitative and qualitative data are combined in the “Discussions” section below.

## Results

### Chinese English as a Foreign Language Learners’ Level of Flow

**FIGURE 3:**
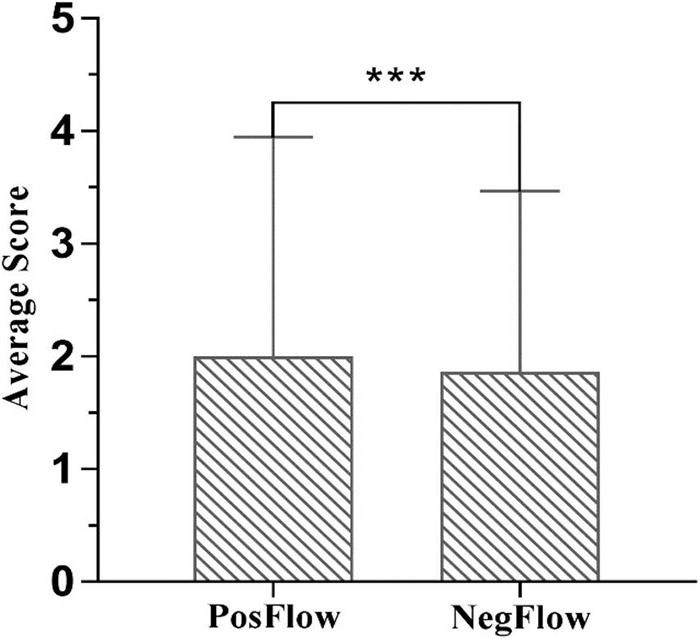
A comparison of average PosFlow and NegFlow scores.

Our first research question addressed how much flow Chinese EFL learners experienced in a blended learning environment. The researcher calculated the mean scores of PosFlow (Cronbach’s α = 0.893; *Enjoyment dimension*) and NegFlow (Cronbach’s α = 0.793; *Boredom* and *Anxiety dimensions*) in the FLFS. As shown in Figure 3, Chinese EFL learners experienced much more positive flow (*M* = 30.60, *SD* = 5.65; nine items) than negative flow (*M* = 15.04, *SD* = 3.65; five items). Their average of means were 3.40 (*SD* = 0.63) and 3.008 (*SD* = 0.73).

### The Relationship Between Positive Flow and Negative Flow

The second research question examined the relationship between positive flow (PosFlow) and negative flow (NegFlow) in a blended learning environment. A significant difference was observed (*df* = 606, *t* = 59.54, *p* < 0.001^***^, 95% CI [15.05, 16.08], effect size *r*^2^ = 0.854, Cohen’s *d* = 4.84) from a paired-sample *t* test and there was a large effect size (Plonsky and Oswald, 2014). Surprisingly, further analysis revealed a weak, but significant positive correlation between positive flow and negative flow (*r* = 0.092*) (Cohen, 1988), indicating the two sides of flow slightly overarched and are not situated in extremes of a continuum.

### Effects of Learner-Internal and Teacher-Related Variables on Flow

Research Question 3 examined the associations between predicting variables and positive and negative flow. As a first step, the researcher conducted Pearson correlation analyses to identify significant relationships (see Table 2). Based on the correlations, the second step involved two stepwise linear regression analyses where independent variables that were linked significantly (*p* < 0.05*, 0.01^**^, and 0.001^***^) with the dependent variables were included. As a result, the researcher not only identified the varying effects of all independent variables on flow, but also the strongest predictors of PosFlow and NegFlow.

**TABLE 2:** Inter-correlations between independent variables and regressions predicting PosFlow and NegFlow.

Variables	PosFlow									NegFlow								
	Correlation	Fit Index			Coefficient			Collinearity Statistics		Correlation	Fit Index			Coefficient			Collinearity Statistics	
	ρ*/r*	*R* ^2^	*R^2^Adjusted*	*F*	*B*	β	*t*	VIF	DW	ρ*/r*	*R* ^2^	*R^2^Adjusted*	*F*	*B*	β	*t*	VIF	DW
**Gender**	0.074	0.398	0.393	79.353***					1.929	–0.010	0.077	0.073	16.873***					1.866
**Major**	0.094*									0.178***				1.738	0.183	4.547***	1.056	
**Age**	–0.124**									–0.133**				–0.609	–0.154	–3.852***	1.042	
**YSLE**	0.069									–0.010								
**GKE**	0.137**									0.047								
**FS**	0.115**									0.114**				0.017	0.125	3.125**	1.052	
**SPEP**	0.271***				0.496	0.167	4.717***	1.257		0.060								
**FLM**	0.340***									0.053								
**SAP**	0.386***									0.019								
**ATFL**	0.531***				2.212	0.339	8.923***	1.439		0.037								
**SFTU**	0.371***				1.237	0.182	5.261***	1.194		0.029								
**Writing**	–0.068									–0.103*								
**Reading**	0.016									0.078								
**Listening**	0.021									0.025								
**Speaking**	–0.025									–0.068								
**Translation**	0.012									0.053								
**ATFLT**	0.382***				1.198	0.162	4.48***	1.312		0.028								
**TFFLU**	0.261***				0.572	0.099	2.931**	1.141		0.038								
**TP**	0.267***									–0.018								

***p < 0.001, **p < 0.01, *p < 0.05.

*ρ Refers to Spearman correlation coefficients (The Spearman correlation (ρ) between two variables is equal to the Pearson correlation (r) between the rank values of those two variables. The difference is that the former assesses monotonic relationships (whether linear or not), while the latter assesses linear relationships. In this study, the researcher used the Spearman correlation analysis to determine the relationship between gender or major and other variables, and the Pearson correlation analysis to examine the correlations between other variables.) for Gender/Major and r refers to Pearson correlation coefficients for all other variables, B refers to Unstandardized Coefficients, β refers to Standardized Coefficients, DW refers to Durbin–Watson test.*

#### Leaner-Internal Variables

Two independent *t*-tests indicated that there were no significant differences between 383 male participants and 224 female participants for both PosFlow (Males *Mean* = 30.36, *SD* = 5.86; Females *Mean* = 31.02, *SD* = 5.26) and NegFlow (Males *Mean* = 15.05, *SD* = 3.66; Females *Mean* = 15.02, *SD* = 3.65) (see Table 3). Another round of independent *t*-tests revealed that 498 natural science majors exhibited a marginally significant difference from 109 social science majors on PosFlow (NS *Mean* = 30.81, *SD* = 5.59; SS *Mean* = 29.66, *SD* = 5.86; *df* = 605, *t* = 1.927, *p* = 0.054, *r*^2^ = 0.061, *Cohen’s d* = 0.157) with a medium effect size, while there was a significant difference on NegFlow (NS *Mean* = 15.35, *SD* = 3.62; SS *Mean* = 13.61, *SD* = 3.49; *df* = 605, *t* = –4.568, *p* < 0.001, *r*^2^ = 0.033, *Cohen’s d* = 0.371) with a medium effect size (see Table 3).

**TABLE 3:** Overview of the effects of the independent variables on PosFlow and NegFlow.

Variables	PosFlow					NegFlow			
	*df*	*F*/*t*	*P*	*r*^2^/eta^2^	*Cohen’s d/f^2^*	*F*/*t*	*p*	*r*^2^/eta^2^	*Cohen’s d/f^2^*
**Gender**	605	1.399	ns			0.089	ns		
**Major**	605	1.927	0.054	0.006	0.157	–4.568	***	0.033	0.371
**Age**	6, 600	2.671	*	0.026	0.027	5.898	***	0.056	0.059
**YSLE**	18, 588	1.688	*	0.049	0.052	0.709	ns		
**GKE**	2, 604	27.908	***	0.085	0.093	1.529	ns		
**SPEP**	2, 604	34.993	***	0.104	0.116	0.159	ns		
**FS**	97, 300	1.133	ns			1.204	ns		
**FLM**	3, 603	26.355	***	0.116	0.131	0.447	ns		
**SAP**	4, 602	25.583	***	0.150	0.176	3.789	**	0.025	0.026
**ATFL**	4, 602	60.504	***	0.287	0.403	0.544	ns		
**SFTU**	4, 602	26.643	***	0.150	0.176	0.845	ns		
**Writing**	2, 604	8.484	***	0.072	0.076	6.171	**	0.020	0.020
**Reading**	2, 604	0.442	ns			4.000	*	0.013	0.013
**Listening**	2, 604	1.496	ns			0.225	ns		
**Speaking**	2, 604	0.793	ns			2.974	0.052	0.010	0.010
**Translation**	2, 604	0.523	ns			0.217	ns		
**ATFLT**	3, 603	36.603	***	0.154	0.182	0.311	ns		
**TFFLU**	4, 602	12.199	***	0.075	0.081	0.722	ns		
**TP**	4, 602	12.348	***	0.076	0.082	1.086	ns		

****p < 0.0001, ***p < 0.001, **p < 0.01, *p < 0.05.

*YSLE, years spent in learning English; GKE, English score in Gaokao; SPEP, self-perceived English proficiency; FS, score in the final exam; FLM, mastery of foreign language; SAP, standing among peers; ATFL, attitude toward the foreign language; SFTU, students’ familiarity with technology use; ATFLT, attitude toward the foreign language teacher; TFFLU, teacher’s frequency of FL use; TP, FL teacher predictability; the effect of gender is determined on t, r^2^, and Cohen’s d, the effect of other variables is determined on F, eta^2^, and Cohen’s f^2^.*

**FIGURE 4:**
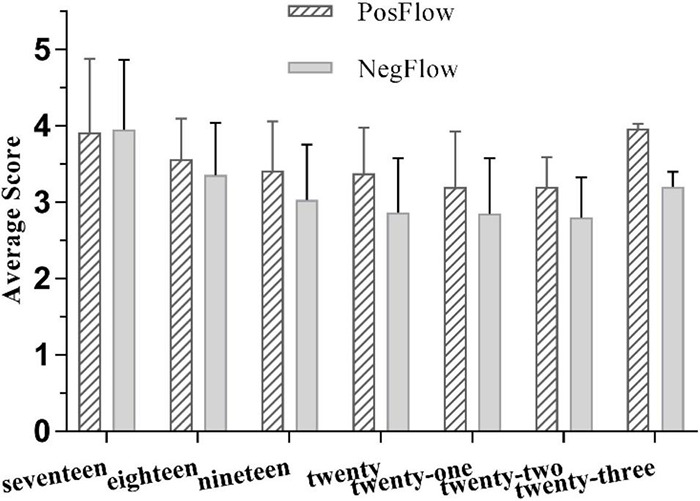
The effects of age on PosFlow and NegFlow (Means and SD).

One-way ANOVAs revealed that age had a small effect on PosFlow and a small-to-medium effect on NegFlow (see Table 3; Plonsky and Oswald, 2014). In view of the differences in the number of items in the 5-Likert FLFS (nine items for PosFlow and five items for NegFlow), the average scores of means of PosFlow and NegFlow were used for comparison (see Figure 4). Generally, Chinese EFL students experienced higher levels of positive flow (average means ranging from 2.963 to 3.917) than negative flow (average means ranging from 2.8 to 3.95). Specifically, younger participants reported greater positive and negative flow than their elderly counterparts (PosFlow Means: 17-year-old group = 35.250, 23-year-old = 26.667; NegFlow Means: 17-year-old group = 19.75, 23-year-old = 16.00), although there was no significant difference between the two age groups. Interestingly, the younger group of 17-years-olds reported higher levels of boredom and anxiety than enjoyment, suggesting that immature language learners are more susceptible to challenges when performing FL tasks.

One-way ANOVAs showed that years spent in learning the FL, English scores in *Gaokao*, and students’ self-perceived language proficiency all had significant effects on PosFlow and small to large effect sizes^4^ were observed (Cohen, 1988), but had no effect on NegFlow (see Table 3). Furthermore, the English final exam taken at the end of the most recent semester had no effect on both PosFlow and NegFlow (see Table 3). To examine the significant differences between different groups, three achievement groups (i.e., low, middle, and high achievement groups) were created based on participants’ average English test scores in *Gaokao* and standard deviation, following the practice of Li et al. (2020). Participants in the low achievement group received English test scores which were more than one standard deviation below the mean, those in the middle achievement group scored within one standard deviation below and above the mean, and those in the high achievement group had scores above one standard deviation above the mean. This pattern was followed when participants were divided into three groups based on their self-perceptions of language proficiency. The detailed information about the *Gaokao* English test scores and students’ self-perceived language proficiency scores of the whole sample are presented in Table 1, and the distribution of participants in the three groups is presented in Appendix. *Post hoc* Gabriel tests^5^ revealed significant differences in PosFlow between the three achievement groups in *Gaokao* (*p* < 0.01 or.001) and self-perceived language proficiency (*p* < 0.01, 0.05, or 0.001, respectively).

**FIGURE 5:**
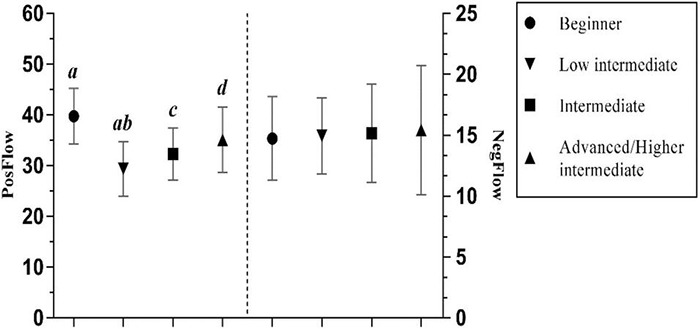
The effects of FL mastery on PosFlow and NegFlow (Means and Significant Differences).

One-way ANOVAs showed a significant effect of overall mastery of FL on PosFlow rather than NegFlow with a medium effect size (Cohen, 1988; see Table 3). Because only one participant rated himself as “advanced”, a single level was created (“advanced/high intermediate”). The mean score on the 4-point Likert scale was 2.35 (*SD* = 0.763). *Post hoc* Gabriel tests revealed significant differences on PosFlow between beginners or low intermediate, and intermediate and advanced/higher intermediate FL learners (all *p* < 0.001), while there was a significant difference between intermediate and advanced/higher intermediate (*p* < 0.05) (see Figure 5). A noteworthy finding was that advanced and higher intermediate students experienced both the most enjoyment as well as boredom and anxiety, indicating that competent FL learners tend to undergo negative emotions when the FL tasks are either too easy or too demanding.

**FIGURE 6:**
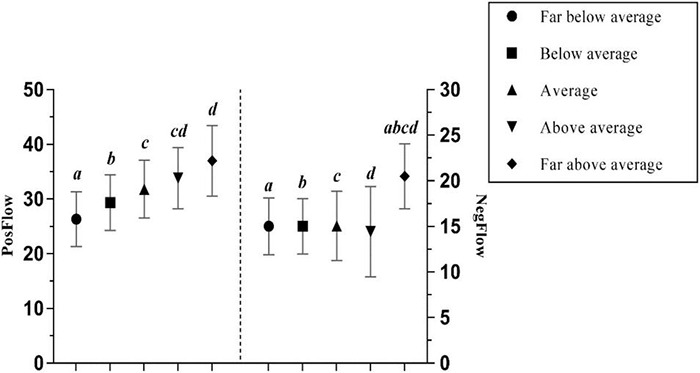
The effects of standing among peers in the FL class on PosFlow and NegFlow (Means and Significant Differences).

One-way ANOVAs indicated that standing among peers had a significant positive effect both on PosFlow – an effect size between small and large – and on NegFlow – a medium effect size (Cohen, 1988; see Table 3). Values of both positive and negative flow peaked at learners who were far above average, whereas they gradually declined in “far below average” FL learners. *Post hoc* Gabriel tests showed that significant differences occurred in PosFlow between those who achieved “far above average” or “above average” from those who achieved “far below average” and those who achieved “below average” respectively (all *p* < 0.001), as well as between the “far above average” and the “average” (*p* < 0.05) FL learners, while statistically significant differences were observed in NegFlow between the “far above average” and all other groups (all *p* < 0.001) (see Figure 6). Interestingly, outstanding FL students are more likely to feel bored when given relatively easy FL tasks, and they may also grow anxious when they realize they need to maintain their top position.

**FIGURE 7:**
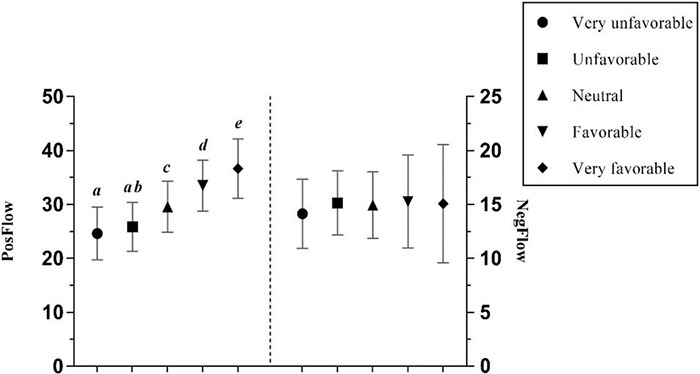
The effects of students’ attitude toward the FL on PosFlow and NegFlow (Means and Significant Differences).

The attitude toward the FL was found to have a significant positive effect on PosFlow with a large effect size, and no significant effect on NegFlow (Cohen, 1988; see Table 3). Specifically, Chinese EFL learners were more likely to experience positive flow when they held a very favorable attitude toward the language, while negative flow experiences were rare in learners with very favorable attitudes. *Post hoc* Gabriel tests revealed significant differences in PosFlow between most groups (all *p* < 0.001), except for the relationship between the very unfavorable and unfavorable FL learners (*p* = ns). Figure 7 illustrates those differences.

**FIGURE 8:**
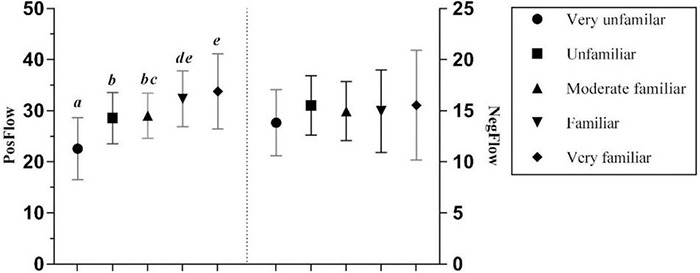
The effects of students’ familiarity with technology use on PosFlow and NegFlow (Means and Significant Differences).

Learners’ familiarity with the use of technology in the FL class significantly and positively affected the intensity of PosFlow with a borderline large effect size (Cohen, 1988), but NegFlow was unaffected (see Table 3). *Post hoc* Gabriel tests revealed significant differences in PosFlow between those very unfamiliar with technology use and the other groups (all *p* < 0.001) (see Figure 8). Surprisingly, with increased familiarity with technology use in the FL class, the values of both positive and negative flow increased, indicating those FL learners who were (very) familiar with the use of technology reported as much enjoyment as boredom and anxiety. A possible explanation could be that Chinese EFL learners were enjoying learning English online, but also felt overwhelmed by the challenges associated with using new software, applications, and/or smart devices.

#### Teacher-Related Variables

There was a complex relationship between the proportion of time FL teachers spent instructing their students in writing, reading, listening, speaking, and translating, and their FL students’ levels of positive and negative flow. Accordingly, three groups (i.e., low, middle, and high level of involvement groups) were created based on values within the lower, middle and upper 33.33% brackets respectively. The researcher observed a significant effect of writing on PosFlow with a medium effect size, while there was a significant difference between these groups on NegFlow with a small effect size (see Table 3). The results indicated that the more FL teachers instructed learners to improve their writing, the less likely they were to become bored or anxious (NegFlow: Mean for the high involvement group = 14.55; Mean for the low involvement group = 15.88) while performing these tasks in a blended learning environment.

**FIGURE 9:**
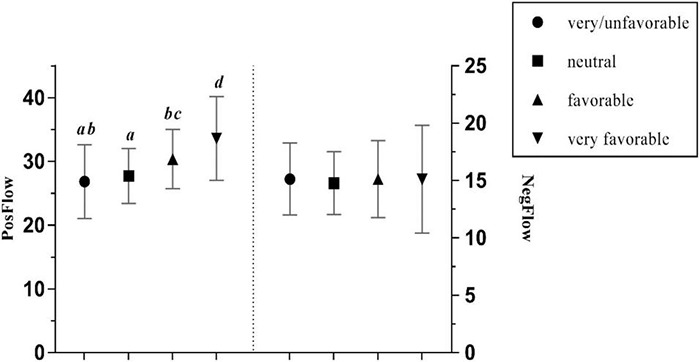
The effects of students’ attitude toward the FL teacher on PosFlow and NegFlow (Means and significant Differences).

The attitude toward the FL teacher was found to have a significant positive effect on PosFlow with a large effect size, but not on NegFlow (Cohen, 1988; see Table 3). Because only a few participants rated themselves as “very unfavorable” toward their FL teacher, a single level was created (“very/unfavorable”). The mean score on the 4-point Likert scale was 3.01 (*SD* = 0.766). Generally, more positive attitudes were associated with higher levels of positive flow, whereas FL learners with negative attitudes were less likely to experience positive flow, but were more likely to experience boredom and anxiety. Unsurprisingly, *post hoc* Gabriel tests indicated that FL learners with very favorable attitudes toward their FL teacher reported significantly higher level of PosFlow than all other groups (all *p* < 0.001), while those with very unfavorable attitudes experienced significantly lower level of PosFlow (*p* < 0.001) and a larger but not statically significant level of NegFlow (see Figure 9).

**FIGURE 10:**
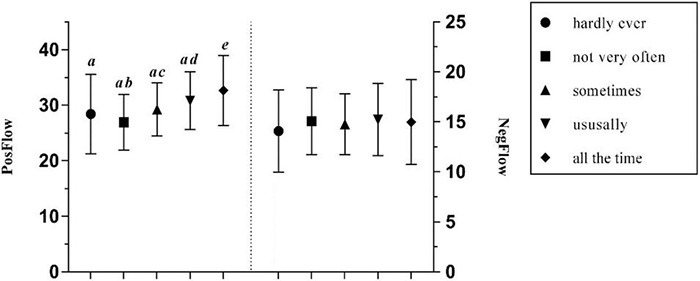
The effects of teacher’s frequency of FL use on PosFlow and NegFlow (Means and significant Differences).

One-way ANOVAs revealed that there was a significant difference between how frequently a teacher used the FL in a blended learning environment and PosFlow (all *p* < 0.001; see Table 3) with a medium effect size, but its effect on NegFlow was not statistically significant. *Post hoc* Gabriel tests showed that when FL teachers used the target language all the time, their students were significantly more likely to experience positive feelings than other groups (all *p* < 0.001 or 0.01), while a teacher who hardly ever used the FL language in class would be more likely to provoke negative emotions in their students (see Figure 10).

**FIGURE 11:**
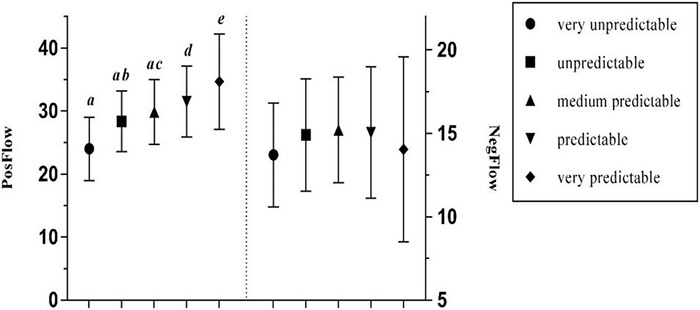
The effects of teacher predictability on PosFlow and NegFlow (Means and Significant Differences).

It was found that teacher predictability had no effect on NegFlow, but a significant positive effect on PosFlow – a medium effect size (Cohen, 1988; see Table 3). Further *post hoc* Gabriel test on participants’ level of positive flow showed that there were significant differences between students with a predictable or very predictable FL teacher and all other groups (see Figure 11). More specifically, learners who perceived their teacher as very predictable were most likely to experience positive flow (*Mean* = 34.677, *SD* = 7.582), which gradually declined at those with very unpredictable teachers (*Mean* = 24.00, *SD* = 5.011). Surprisingly, learners who rated their teacher to be medium predictable reported the highest level of negative flow (*Mean* = 15.207, *SD* = 3.157).

### The Strongest Co-predicting Variables on Flow

In order to identify the best co-predictors of PosFlow and NegFlow, the researcher conducted two stepwise multiple regression analyses where only independent variables that showed significant correlations with PosFlow and NegFlow entered. These variables included learner-internal and teacher-related variables (see Table 2). In addition, all values for the variance inflation factor (VIF), which examined the severity of multicollinearity, were around 1, suggesting that there is no multicollinearity problem (Kutner et al., 2004). Further, results for the Durbin-Watson tests were all around 2 (PosFlow = 1.929, NegFlow = 1.866), indicating that there is no auto-correlation in the residuals between first-order variables. In the end, the researcher identified significant regression equations for PosFlow and NegFlow respectively (see Table 2). Generally, five significant predictors emerged for PosFlow and three for NegFlow. More specifically, the strongest predictor of PosFlow was learners’ attitude toward the FL (β = 0.339), followed by students’ familiarity with technology use in class (β = 0.182), their self-perceived language proficiency (β = 0.167), and attitude toward the FL teacher (β = 0.162), with the weakest variable being teacher’s frequency of FL use (β = 0.099), while the best predictor of NegFlow was participants’ major (β = 0.183), followed by students’ age (β = –0.154), with the least contribution from their scores on the final English exam (β = 0.125).

### Selection of Participants’ Views on Episodes of Positive and Negative Flow in the Chinese Blended English as a Foreign Language Classroom

The qualitative data was gathered through an open question at the end of the questionnaire. The researcher used a refined approach to examine the entries where a *theme* in the form of “a simple sentence, a string of words with a subject and a predicate” (Berg and Lune, 2012, p. 359) was chosen as a unit of analysis. To illustrate how the data was categorized and coded, Example 1 displayed a complete feedback entry from a participant named YSQ (male, 19).

**Example 1: Feedback entry.**

^1^When watching video and listening to audios via the Internet, I felt great convenience in getting access to lecture resources. ^2^And there is no time limit completing a task. ^3^However, with a variety of learning materials flooding in, my incompetence in language use made it hard for me to follow what was moving on at a high speed. ^4^Thus, it seems impossible to concentrate on learning task, which led to low efficiency in completing listening and speaking tasks. ^5^In comparison, the traditional classroom learning was filled with teacher-learner and peer interactions, which ultimately stimulated the potential for and enhanced the confidence in learning.

It is worth noting that in Example 1, sentence 2 was factual and unconnected to the participant’s feelings in a blended EFL learning context. As a result, this sentence was removed from further analysis, and the remaining sentences were classified as to whether they indicated positive or negative flow experiences. We next categorized data into discrete dimensions developed in a prior study conducted in a blended learning environment by Wang and Huang (2022) using descriptive themes and codes, as shown in Table 4.

**TABLE 4:** Example of the coding process.

Theme	Categories	Dimensions	Descriptions/Codes
^1^When watching video and listening to audios via the Internet, I **felt great convenience** in getting access to lecture resources.	PosFlow	*FLF-Enjoyment*	Perception of easy access to learning resources
^3^However, with a variety of learning materials flooding in, my **incompetence in language use** made it **hard for me to follow** what was moving on at a high speed.	NegFlow	*FLF-Boredom*	Feeling of insufficient language skills
^4^Thus, it seems **impossible to concentrate on learning task**, which led to **low efficiency** in completing listening and speaking tasks.	NegFlow	*FLF-Anxiety*	Perception of failure caused by a lack of attention
^5^In comparison, the traditional classroom learning was filled with **teacher-learner and peer interactions**, which ultimately **stimulated the potential** for and **enhanced the confidence** in learning.	PosFlow	*FLF-Enjoyment*	Perception of achievement related to interaction with teacher or peers

*The superscript numbers “1, 3, 4, 5” (“2” is eliminated from the further coding process) refer to the sequence of sentences in Example 1: Feedback entry. PosFlow, positive flow; NegFlow, negative flow; FLF, foreign language flow.*

Since the qualitative data is primarily intended to provide examples of flow, the researcher selected excerpts from participants based on these themes and codes, as well as their accompanying predicative variables.

#### Convenient Access to Learning Resources Contributes to a Greater Sense of Confidence

One participant, LYT, mentioned the impact that blended EFL learning had on her confidence:

LYT (female, 19): “I can obtain learning materials via online apps and watch TV series such as Big Bang, Friends and Two Broke Girls It’s very convenient because I can have more time to practice and complete my tasks and I think this will activate my learning potential. It has also increased my confidence in my ability to learn English.”

#### A Blended Learning Environment Fosters Interest in FL Learning, Which Facilitates Positive Flow in Blended Learning

At some point, EFL learners like WYY realized that:

WYY (female, 19): “I think the mixed teaching approach is better and I like it. I find it very interesting that the teacher incorporated some short videos and recordings into the course. We can see different answers on *Danmupai*, which is a very useful feature.”

#### Blended Learning Is Intriguing and Enjoyable

In blended learning, flow can occur when learners find the process enjoyable, as described by SWH:

SWH (male, 19): “The teaching activities of blended teaching approach are very enjoyable. Online instruction gives us ample time to learn. It is more flexible than traditional classroom instruction.”

#### Interest in a Blended Learning Environment Is the Basis for Openness to New Knowledge

English as a foreign language learners will be more likely to absorb new information if they perceive a positive learning environment and subsequently develop a strong interest in the subject. JYC recalled his positive experience in a blended learning environment:

JYC (male, 18): “The blended teaching approach can easily arouse students’ interest in the curriculum. And the classroom atmosphere will also become more relaxed and pleasant. We are better prepared to receive new knowledge.”

#### Teacher-Induced Positive Flow

The teacher plays a crucial role in facilitating class interactions and thereby creating a positive learning atmosphere, especially in traditional FL classes. SQH reported his case as follows:

SQH (male, 19): “I cannot concentrate for too long and I cannot communicate with the teacher immediately during online courses. However, I am able to interact with the teacher and my learning efficiency increases in the classroom.”

#### Negative Flow Is Not Necessarily Hindered by Blended Learning

Though blended learning encourages learners to focus on the task at hand, EFL learners can be distracted from what they are doing, as LQT and CZY described:

LQT (female, 19): “The mixed teaching approach can improve our English in all respects. But sometimes online activities are too many and they will distract us from the course.”

CZY (male, 18): “Online courses offer greater flexibility. But we may be distracted by some activities. I think the mixed teaching method is better for our studies.”

#### Learners Are Anxious and Nervous Due to a Lack of FL Proficiency

Learning English as a foreign language is more challenging and anxiety-provoking when one does not possess a good command of the target language. DJJ’s description illustrated this well:

DJJ (male, 19): “I need to be mindful of the teacher’s message when I’m using *Danmupai* because I have to wait until I have the chance to answer the question. Normally, I can’t be so engaged in the classroom.”

#### Boredom Results From Loss of Concentration

A state of negative flow is created when EFL learners lose interest in technology-intensive tasks. WCL shared her experience in this regard:

WCL (female, 18): “I think the blended teaching approach is better. Online education can arouse my interest, but it does not last long. And I cannot concentrate very long, and it becomes boring afterward. Classroom teaching is clearer and easier to understand.”

## Discussion

Overall, the purpose of the present study was to examine the juxtaposition of PosFlow and NegFlow, and to determine the effects of learner and teacher variables on both constructs within a blended Chinese EFL context. This study has laid a solid foundation for the conceptual and empirical inquiry into the dynamics of flow, emphasizing the necessity of incorporating both positive and negative flow into a single study by identifying the strongest predictive variables for PosFlow and NegFlow separately, and demonstrating the complex dynamic interactions between the two.

The first research question examines the level of flow among Chinese EFL learners in a blended learning environment. Results indicate that Chinese EFL learners generally experience more positive flow than negative flow, indicating a tendency for them to be engaged and satisfied with their FL classes as opposed to feeling bored or anxious about them. The findings, however, contradict what was revealed in Wang and Huang’s (2022) study, which indicated much negative flow than positive flow. In fact, the contradiction corroborates the claim that the intensity of language anxiety and boredom can change in a virtual environment (Wang and Derakhshan, 2021), indicating that learners’ levels of flow are affected by a range of factors, including both learner-internal and learner-external factors, as well as their learning environments.

The answer to the second research question reveals a significant, but weak positive correlation between PosFlow and NegFlow, which corroborates earlier findings by Dewaele and MacIntyre (2019) which indicated that the two sides of flow share a very limited amount of variance. Qualitative findings also demonstrate a complex correlation between PosFlow and NegFlow, suggesting that despite the fact that EFL students are attentive and enjoy FL tasks in a blended environment, they may also experience tension and distraction, as illustrated by DJJ’s comment, “I need to be mindful of the teacher’s message when I’m using *Danmupai* because I have to wait until I have the chance to answer the question. Normally, I can’t be so engaged in the classroom”. Both quantitative and qualitative findings indicate that positive and negative flow can coexist in a blended learning environment which fosters both interest and confidence in FL learning. Naturally, when FL learners perceive blended learning to be compelling, they are more inclined to concentrate on the FL tasks, which leads to a virtuous circle where task complexity is appropriate and learners are actively participating. Thus, this increases learners’ task engagement and may, as a result, reduce the likelihood of negative experiences among learners, which is consistent with what SWH has stated, “The teaching activities of blended teaching approach are very enjoyable. Online instruction gives us ample time to learn. It is more flexible than traditional classroom instruction.” However, highly motivated students may occasionally feel nervous when they place high expectations on external rewards.

The third research question examines the predicting variables from two perspectives: their effects on flow (PosFlow and NegFlow) and their varying predictive effects. On the one hand, Table 3 provides a quick overview of the effects of different variables on flow, and demonstrates that learner-internal variables appear to be more strongly associated with both positive and negative flow than the teacher-related variables. Indeed, learner variables explain a larger proportion of the variance in positive and negative flow. On the other hand, multiple regression analyses allow us to identify the strongest predictors of PosFlow and NegFlow. Dovetailing with the findings of Dewaele and MacIntyre (2019), this study confirms that the positive and negative aspects of flow, though coexisting dynamically, are not identical, since independent variables operate differently to predict positive and negative flow. Of note, there was no variance explained by both PosFlow and NegFlow in the predicative model.

Furthermore, the findings support the assertion in Dewaele et al.’s (2018) study that teacher predictability has no effect on the negative emotion, such as foreign language anxiety, but a significant positive effect on the positive emotion, such as foreign language enjoyment. Generally, learners who perceive their FL teacher as very predictable tend to experience most positive flow (*Mean* = 34.677). However, it is striking that students with medium predictable teachers are most likely to experience negative flow (*Mean* = 15.207), as opposed to those with very unpredictable teachers (*Mean* = 13.70), though *post hoc* tests did not demonstrate a significant difference between the two groups. One possible explanation could be that FL learners can access a wide range of learning resources in a blended environment without having to rely on face-to-face instruction from a teacher. Therefore, the teacher has less control over the students’ emotional state. Moreover, these findings are consistent with literature which has demonstrated that learners with very favorable attitudes toward the FL experienced greater enjoyment than those with very unfavorable attitudes (Dewaele et al., 2018), and that those with favorable attitudes experienced less boredom and anxiety.

However, not in line with what has been revealed by Dewaele et al. (2018) and Dewaele and MacIntyre (2019) who asserted that students’ relative standing among peers only have a significant effect on PosFlow rather than on NegFlow, the current study reveals its significant effects on both PosFlow and NegFlow. More specifically, FL learners’ level of flow increases at higher relative standing among peers (Dewaele and MacIntyre, 2019), and that the awareness of a higher peer standing is more likely to result in enjoyment (Dewaele and MacIntyre, 2014), a major positive emotion that has some commonalities with positive flow (Dewaele and MacIntyre, 2019) and is a fundamental component of flow in both a blended (Wang and Huang, 2022) as well as a technology-based learning environment (Schüler, 2012; Li R. et al., 2021; Liu and Song, 2021). Surprisingly, students who rated themselves as far above average experience the highest levels of both positive (*Mean* = 37.00) and negative (*Mean* = 20.50) flow, suggesting that FL learning is so demanding that even highly competent students can become bored or even anxious when a balance between challenge and skills is distorted.

Moreover, the finding that advanced or higher intermediate FL learners are more likely to experience much more positive emotions than beginners partially mirrors the elucidation of FLE in Dewaele and MacIntyre’s (2014) and Dewaele et al.’s (2018) studies as well as the interpretation of PosFlow in Dewaele and MacIntyre’s (2019) study. Surprisingly, the present study shows a concomitant boost in PosFlow and NegFlow among advanced or higher intermediate FL learners, contradicting the finding from Dewaele et al. (2018) that more advanced FL learners felt substantially less anxiety and more enjoyment. A plausible interpretation is that though experienced FL learners easily develop positive self-images and FL attitudes that intrinsically motivate them to perform better (Dörnyei and Ryan, 2015), this may contribute to their high expectations on academic performance. As a result, when these expectations are not met, they may become bored, anxious, or even dissatisfied with the FL tasks.

Another striking finding in the present study is that familiarity with technology use is significantly correlated with learners’ tendency to experience positive flow. Naturally, learners vary in their willingness and ability to use technology in their FL learning. In fact, learners who are (very) familiar with technology use are more likely to focus on tasks and enjoy tackling those tasks, thereby becoming motivated and more engaged in blended learning tasks (Albiladi and Alshareef, 2019), and subsequently developing a positive attitude toward the FL learning (Pan, 2020; Rafiee and Abbasian-Naghneh, 2021).

Furthermore, when both PosFlow and NegFlow are considered together, it is noteworthy to find that learner-internal variables explain 36.3% of the variance in PosFlow but only 7.7% of the variance in NegFlow. The relatively weak contributions of learner variables to NegFlow have not been discussed previously, but in this study it can be attributed to the strengths of blended learning where activities tend to “motivate(s) learners to interact and engage in the language learning process” (Albiladi and Alshareef, 2019, p.234), and learners are able to develop a positive attitude toward the FL and to perceive enjoyment in the blended EFL learning (Davis et al., 1992; Yoon and Lee, 2010; Akbarov et al., 2018). Interestingly, no teacher-related variables enter the NegFlow regression model, while only two teacher variables enter the PosFlow regression model. The researcher attributes the imbalanced contribution of the learner and teacher variables in the regression model to the unique characteristics of blended learning, which is considered as an effective approach that fosters motivation and autonomy in FL learning (Banditvilai, 2016; Akbarov et al., 2018) in flexible and meaningful ways (Senffner and Kepler, 2015). Additionally, EFL students would also benefit from blended learning (Banditvilai, 2016), as it allows them to choose learning material at random and learn for a specific period of time, regardless of whether or not they are in a classroom milieu. Therefore, when learning autonomy is established, the teacher will recede into the shadow of motivated behaviors on the part of the students.

## Limitations, Suggestions for Future Research, and Pedagogical Implications

Though the present study provides new insights into flow in a blended EFL learning context, there are several methodological limitations to its research design. Firstly, the researcher has indicated that the study is based on convenience samples from a specific educational context in China. Convenience sampling is a preferred sampling strategy in social sciences due to its accessibility and proximity to participants. However, researchers must be aware of the limitations of convenience sampling when interpreting results since it lacks generalizability and may result in a biased sample (Jager et al., 2017). In this regard, our sample may not fully represent the target population of Chinese EFL learners. Secondly, there was a notable disproportion between the number of participants majoring in natural science subjects and social science subjects (82.04% versus 17.96%), which may lead to an inaccurate estimation of Chinese students’ overall level of flow. Thirdly, the study did not examine the relationship between learners’ level of flow and FL achievement, leaving room for future research. Lastly, the feedback corpus only provided examples to illustrate the quantitative findings, but did not provide comprehensive qualitative analysis.

Accordingly, the researcher recommends that future research should take into account the following factors. In the first place, homogeneous convenience sampling tends to overcome the disadvantages of conventional sampling and is more generalizable. Second, since major has been found to influence negative flow, future studies may benefit from the expansion of social science majors outside of a single educational setting and verify the validity of the major-flow association. Third, prospective studies should incorporate participants’ academic achievement into the correlation analysis and explore how PosFlow and NegFlow might be used to predict the success or failure of FL learning. Last but not least, researchers should consider performing a Pearson Chi-square analysis based on the qualitative data in order to determine the frequency of flow-related categories.

Toward reinforcing the pedagogical implications that have been discussed throughout the paper, the researcher suggests that both positive and negative states of flow be examined together in a single study. As the learners’ attitude toward the FL accounts for the greatest variance in the regression analysis, it seems necessary to use blended learning to cultivate a positive L2 image and motivate FL learners to engage in FL activities. Furthermore, as Chinese EFL learners attach great importance to their academic position within peers and their academic performance is heavily influenced by teacher appraisal (Wang X. et al., 2021) even when they are approaching self-autonomy in a blended EFL learning environment, FL teachers are reminded to inspire higher intermediate, if not advanced, FL learners by acknowledging their previous achievements, and, at the same time, not to demotivate the beginners by underestimating their competence. Last but not least, regarding the discrete language skills, the significant positive relationship between writing and both positive and negative flow, as well as the significant negative relationship between reading and negative flow, suggests that FL teachers should accurately evaluate the language proficiency of their students to mitigate their possible negative interpretations of FL courses, especially when such activities are involved.

## Data Availability Statement

The raw data supporting the conclusions of this article will be made available by the authors, without undue reservation.

## Author Contributions

XW designed the research, collected and processed the data, and wrote the whole manuscript.

## Conflict of Interest

The author declares that the research was conducted in the absence of any commercial or financial relationships that could be construed as a potential conflict of interest.

## Publisher’s Note

All claims expressed in this article are solely those of the authors and do not necessarily represent those of their affiliated organizations, or those of the publisher, the editors and the reviewers. Any product that may be evaluated in this article, or claim that may be made by its manufacturer, is not guaranteed or endorsed by the publisher.

## Footnotes

1 In this paper, flow refers to a combination of positive flow and negative flow.

2 *Gaokao* refers to the National College Entrance Examination (NCEE), which is a standardized college entrance exam taken on a yearly basis in mainland China. In almost all universities, it is a prerequisite for admission to the undergraduate program.

3 *Wenjuanxing* provides online questionnaire services including questionnaire design, distribution, and analysis. Researchers can include an electronic questionnaire in the website or share a link, or QR codes for participants to complete on their smartphones.

4 Selya et al. (2012) suggest that *Cohen’s f^2^* is appropriate for estimating a variable’s effect size within multivariate regression models where the independent variables are categorical.

5 Field (2013) recommends the use of Gabriel *post hoc* tests when sample sizes are different because they have greater statistical power.

## Appendix

**APPENDIX TABLE:** Participants’ demographic and language-related profile.

Variable	Ranges	Number
Gaokao English test scores	Low achievement group	96
	Middle achievement group	163
	High achievement group	348
SPEP	Low achievers	138
	Middle achievers	59
	High achievers	410
Overall FL mastery	Beginner	82
	Low intermediate	253
	Intermediate	247
	High intermediate	24
	Advanced	1
Standing among peers	Far below average	76
	Below average	193
	Average	276
	Above average	56
	Far above average	6
Familiarity with tech use	Very unfamiliar	13
	Unfamiliar	39
	Moderate familiar	265
	Familiar	234
	Very familiar	56
Attitude toward the FL	Very unfavorable	20
	Unfavorable	66
	Neutral	316
	Favorable	159
	Very favorable	46
Attitude toward the FL teacher	Very unfavorable	2
	Unfavorable	5
	Neutral	154
	Favorable	272
	Very favorable	174
Frequency of FL use by teacher	Hardly ever	21
	Not very often	38
	Sometimes	127
	Usually	282
	All the time	139
The average time in five skills	Reading	26.23%
	Listening	21.86%
	Speaking	14.17%
	Writing	18.11%
	Translating	19.64%
Teacher Predictability	Very unpredictable	10
	Unpredictable	31
	Medium predictable	285
	Predictable	250
	Very predictable	31
